# Optimized Incorporation of Silver Nanoparticles onto Cotton Fabric Using *k*-Carrageenan Coatings for Enhanced Antimicrobial Properties

**DOI:** 10.1021/acsabm.4c01002

**Published:** 2024-09-24

**Authors:** Luana Dumas, Matheus Cardoso de Souza, Elton Guntendorfer Bonafe, Alessandro Francisco Martins, Johny Paulo Monteiro

**Affiliations:** † Laboratory of Materials, Macromolecules and Composites (LAMMAC), Federal University of Technology—Paraná (UTFPR), Apucarana, Paraná 86812-460, Brazil; ‡ Department of Chemistry, Pittsburgh State University, Pittsburgh, Kansas 66762, United States

**Keywords:** bactericidal fabric, kappa-carrageenan, silver nanoparticles, cotton, textile coating

## Abstract

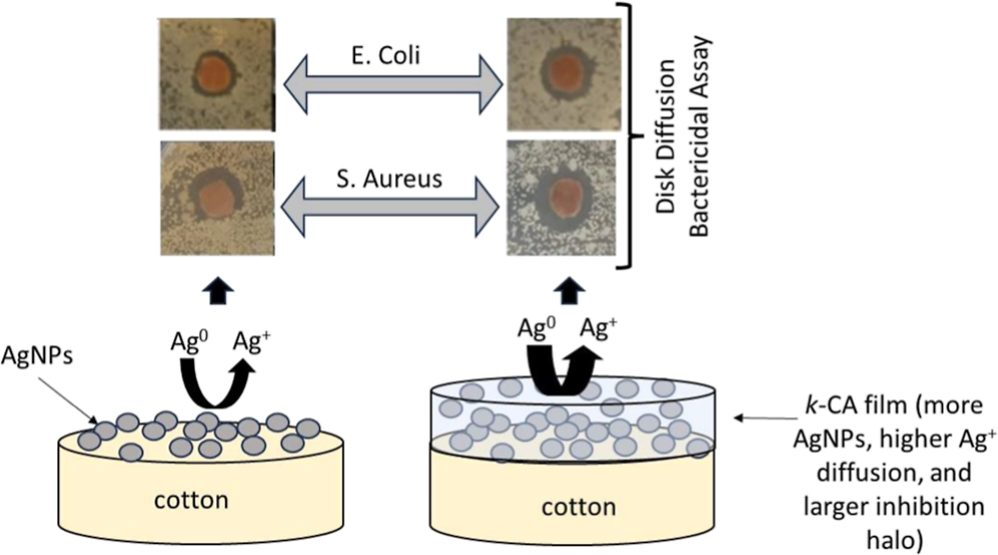

The incorporation of bactericidal properties into textiles is a widely sought-after aspect, and silver nanoparticles (AgNPs) can be used for this. Here, we evaluate a strategy for incorporating AgNPs into a cotton fabric. For this purpose, a bactericidal textile coating based on a composite of AgNPs and kappa-carrageenan (*k*-CA) was proposed. The composite was obtained by heating the silver precursor (AgNO_3_) directly in *k*-CA solution for green synthesis and in situ AgNPs stabilization. Cotton substrates were added to the heated composite solution for surface impregnation and hydrogel film formation after cooling. Direct synthesis of AgNPs on a fabric was also tested. The results showed that the application of a coating based on *k*-CA/AgNPs composite can achieve more than twice the silver loading on the fabric surface compared to the textile subjected to direct AgNPs incorporation. Furthermore, silver release tests in water showed that higher Ag^+^ levels were reached for *k*-CA/AgNPs-coated cotton. Therefore, inoculation tests with the bacteria *Staphylococcus aureus* (SA) using the agar diffusion method showed that samples covered with the composite resulted in significantly larger inhibition halos. This indicated that the use of the composite as a coating for cotton fabric improved its bactericidal activity against SA.

## 1 Introduction

Cotton is a natural textile fiber formed almost exclusively from cellulose,^1^ the most abundant natural polysaccharide on the planet. Cellulose is chemically formed by β-d-glucopyranose units linked by β-1,4-glycosidic bonds.^2^ Cotton has interesting properties such as biodegradability, biocompatibility, hydrophilicity, and chemical and thermal stability, among others.^3^ Many types of research have been carried out to functionalize cotton fibers to incorporate properties such as self-cleaning, antimicrobial activity, ultraviolet (UV) protection, or hydrophobicity.^4^ Finishing on fabric surfaces can be obtained using nanotechnology, and effective results have already been observed even using low concentrations of the nanomaterial.^5^ This advantage is derived from the high surface area/volume ratio.^6,7^

Textiles have been used from garments to medical materials for clinical and surgical purposes.^8−10^ For example, using textiles as dressings has been explored.^11,12^ For this application, the textile material needs to have some specific characteristics such as biocompatibility, nontoxic, nonallergenic, nonadherent to the skin, and antimicrobial properties.^9,10,13^ The cotton properties meet many of these needs. However, natural fibers usually do not have antibacterial action, and the incorporation of this characteristic has been particularly explored.^14,15^ In this sense, metallic nanoparticles such as silver, zinc oxide, and titanium dioxide have been used to provide antimicrobial and antifungal effects to textiles.^10,16^

The silver nanoparticles (AgNPs) synthesis can be achieved through physical, chemical, or green synthesis. AgNPs obtained by physical methods can be through gamma, UV, microwave, and ultrasonic waves.^17^ Arc discharge or cold plasma with spark discharge and pyrolysis has also been used.^18,19^ A silver precursor and a strong chemical reducer (such as sodium borohydride) are used in chemical approach,^20,21^ but the final material is usually cytotoxic.^22,23^ The AgNPs green synthesis,^7,24,25^ on the other hand, avoids the use of toxic chemicals and organic solvents.^26^ It promotes low waste and is a simple, practical, safe, sustainable, and ecologically correct method.^27^ AgNO_3_ bioreducing agents can be plant extracts such as azadirachta leaves,^28^ and natural polysaccharides such as glucose^29^ and carrageenan.^30^ Xanthan, dextran, and chitosan^20^ have also been explored. These biomaterials act as both reducers and stabilizers and can promote antibacterial activity without causing cytotoxicity in the final material.^21^

AgNPs stand out in the development of bactericidal materials. The AgNPs show strong and efficient antimicrobial activities due to their small size, high active surface area, and chemical properties.^31^ The action of AgNPs has been studied against Gram-positive and Gram-negative bacteria,^32,33^ mainly in *Staphylococcus aureus* (SA)^34^ and *Escherichia coli* (EC)*,* which are resistant bacteria found in hospital environments.^35^ The AgNPs action mechanism on bacterial cells normally is through the Ag^+^ ions release due to AgNPs oxidation in aqueous solution.^14,36^ Ag^+^ ions are capable of penetrating or binding the cell wall, and their action can occur mainly through the following pathways: (a) disruption of the cell envelope; (b) generation of reactive oxygen species that interfere with cellular respiration, and (c) interaction with structural molecules in the cytoplasm (such as enzymes and nucleic acid).^9,36,37^ This leads to cell membrane destruction and cell death.^10,31,38^

The association of AgNPs to fabrics has already been widely explored, in which AgNPs can be produced in situ directly on cotton fabric, or they can be synthesized separately and immobilized on it.^9,14,39^ Direct AgNPs immobilization is faster and simpler but has disadvantages such as greater ease of leaching, and mainly, the amount of AgNPs that can be loaded onto the surface is limited to its area. This last aspect will also limit the time that the bactericidal function will be active on the textile and even the intensity of this effect. Polysaccharides have been used to obtain AgNPs in green synthesis. Advantageously, some polysaccharides produce three-dimensional matrices in the form of a hydrogel that can accommodate AgNPs throughout their volume.^40−42^ Hydrogels produced from polysaccharides may be attractive mainly for association with medical textiles as they exhibit interesting properties such as biodegradability, biocompatibility, cytocompatibility, good stability, and low toxicity.^2,43^ These features have been explored in textile engineering and dressing development.^44^

The carrageenans are linear, anionic, and water-soluble polysaccharides. They are formed of sulfated α-1,3 and β-1,4-galactans, and they are obtained from different species of red seaweed.^45,46^ They occur in three distinct chemical compositions based on the number of sulfated groups per disaccharide unit: lambda (λ) (three groups, nongelling), iota (ι) (two groups, weak gelling), and kappa (κ) (one group, strong gelling).^26,47−49^*k*-Carrageenan (*k*-CA) is a polysaccharide from red algae of the Rhodophyceae type,^6,50^ has great gelation capacity, and has been used in AgNPs synthesis and stabilization.^8,19,24,51^ Thus, the *k*-CA hydrogel brings together interesting characteristics and should be a viable alternative to support AgNPs on cotton, but this approach has not yet been reported.

Therefore, we have explored here the green synthesis of AgNPs using *k*-CA. The *k*-CA/AgNPs composite was used to propose a novel cotton coating by its gelation. The coating based on the *k*-CA/AgNPs hydrogel incorporated a stronger antimicrobial and antiadhesive behavior against SA compared to cotton coated only with AgNPs produced directly on the fabric. It was shown that the improved bactericidal properties obtained with the use of the composite were due to the greater capacity to release Ag^+^ ions from the fabric surface. Additionally, the amount of AgNPs incorporated onto the surface was doubled compared to cotton covered only with AgNPs, and this should sustain functionality for longer. Finally, it was shown that the coating based on the use of the *k*-CA/AgNPs composite did not worsen the fibers’ mechanical resistance and did not change the cotton surface wettability. The coating proposed here can be a good alternative for application in technological bactericidal textiles, medical textiles, and wound healing fabrics.

## 2 Materials and Methods

### 2.1 Materials

Commercial *k-*CA (GENUGEL, 277 kDa) formed by altering α-(1,3)-d-galactose-4-sulfated and β-(1,4)-3,6-anydro-d-galactose units^52^ was kindly donated by CP Kelco (Limeira-SP, Brazil). Silver nitrate (AgNO_3_) P.A. (99.0%) was purchased from Sigma-Aldrich (Brazil). Nitric acid HNO_3_ 65% and hydrogen peroxide (H_2_O_2_) 30% were acquired from Anidrol (Brazil). Flat fabric with canvas binding, with 100% cotton composition, and a grammage of 120 g/m^2^, was purchased from the local market (Apucarana-PR, Brazil). The fabric was cut into samples with dimensions of 30 × 10 mm for all analyses except for the tensile strength test, in which the samples were cut in an hourglass shape with a surface area of 90 mm^2^. The materials used were used without prior purification.

Ultraviolet and visible absorption (UV–vis) analyses by surface reflection were performed by using a portable spectrometer coupled with a reflectance accessory (UBS4000, Ocean Optics). Dynamic light scattering analyses (DLS, Litesizer 500, Anton Paar) were performed to determine the hydrodynamic diameter and zeta potential. For this, the mixtures were previously centrifuged for 10 min at 150 rpm. The supernatant was removed and diluted appropriately to maintain a conductivity below 10 μS. Then, an aliquot of the resulting mixture was used for analysis. The dosage of silver in the mixtures was performed by flame atomic absorption spectrometry (FAAS, ICE 3000, Thermo Scientific) using a silver lamp with a wavelength of 328.1 nm and standard aqueous silver solutions (5 mL aliquots) with concentrations of 0.25, 0.8, 3.0, 5.0, 8.0, 12.0, and 15.0 mg·L^–1^ to obtain the calibration curve. The images and elemental surface composition were determined by using a scanning electron microscope (QUANTA-250, FEI) coupled with an energy-dispersive X-ray spectrometer (EDS). The acceleration voltage for image recording was 5 kV, and the samples were previously coated with 10 nm of palladium–gold alloy using sputtering (Sputter coater SCD 050, Baltec). In the tensile test, a universal testing machine (WDW-300E, Time-Shijin Group) was used with a distance between grips of 40 mm and an initial load of 2 N.

To evaluate the bactericidal action, Gram-negative bacteria *E. coli* ATCC 25922 (EC) and Gram-positive *S. aureus* ATCC 25923 (SA) were used for inoculation. Dimethyl sulfoxide (DMSO) (Sigma-Aldrich), Mueller Hinton Agar (BBL), and suspension of *H. pylori* (1 × 108 colony forming units) were used to prepare samples for the analysis of inhibition halo. For the antiadhesive properties, glycerol, Luria–Bertani broth (LB), phosphate-buffered saline (PBS) (pH 7.4), glutaraldehyde (Sigma-Aldrich), sodium cacodylate (Sigma-Aldrich), sucrose (Sigma-Aldrich), and absolute ethanol 99,5% (Anidrol, Brazil) were used.

### 2.2 Methods

#### 2.2.1 Direct AgNPs Synthesis on Cotton

Three fabric samples were immersed in 30 mL of a AgNO_3_ aqueous solution. The system was kept under constant stirring with heating at 80 °C, under reflux, for 120 min. The concentrations of silver precursor tested were 0.5 and 1.0% (wt/v).

#### 2.2.2 Synthesis of Coatings on Cotton Based on *k*-CA and *k*-CA/AgNPs Composite

For *k-*CA-coated cotton preparation, 15 mL of *k*-CA aqueous suspension was prepared under constant stirring and heating at 80 °C, under reflux, for 50 min. Three fabric samples and 15 mL of ultrapure water were added to the *k-*CA suspension and kept under the same reaction conditions for another 120 min for *k-*CA impregnation. The concentrations of *k*-CA tested for the final mixture were 0.5 and 1.0% (wt/v).

For *k*-CA/AgNPs-coated samples obtention, 15 mL of *k*-CA suspensions, prepared as described above, were maintained under reflux at 80 °C. Then, three fabric samples and 15 mL of AgNO_3_ aqueous solution were added to the mixture and kept under the same conditions for 120 min. Two concentrations of *k*-CA and AgNO_3_ were also tested for the final mixture: 0.5 and 1.0% (wt/v).

All synthesis conditions are described in Table 1.

**Table 1:** Concentrations of the AgNO_3_ and *k-*CA Aqueous Solutions That Were Used to Produce Different Coatings on Cotton Fabric Samples

sample	AgNO_3_ (% wt/v)	*k*-CA (% wt/v)
C1	0.0	1.0
C05	0.0	0.5
Ag05	0.5	0.0
A1	1.0	0.0
Ag05C05	0.5	0.5
Ag05C1	0.5	1.0
Ag1C05	1.0	0.5
Ag1C1	1.0	1.0
control	0.0	0.0

All synthesized samples (2.2.1 and 2.2.2 topics) were removed after the time reaction, and the excess solution was removed. Each fabric sample was placed between glass slides and stored at 6 ± 2 °C protected from light for 24 h. Then, the fabric samples were frozen and lyophilized for further analysis. Additionally, 3 mL aliquots of the resulting liquid reaction mixture were stored in eppendorf tubes for further DLS analysis.

#### 2.2.3 Silver Quantification on Covered Cotton Samples

Sample digestion was performed in an acidic medium to determine the amount of inorganic material (in the form of AgNPs) incorporated into the coatings on the fabric samples. For this, the coated and lyophilized fabric samples were immersed in 10 mL of HNO_3_ 65%. The solution was kept at 150 °C in a glycerin bath under a stirring and reflux system for 75 min. Then, the mixture was cooled to 25 °C by using an ice bath, and 5 mL of 30% H_2_O_2_ was added. The mixture was stirred for a further 10 min at 150 °C and finally cooled to room temperature. The final mixture was filtered, and all the contents of the filtrate were used to prepare 50 mL of an aqueous solution. The analysis was performed in triplicate, and the Ag content in the solution was determined by FAAS.

#### 2.2.4 Ag^+^ Release

The coated fabric sample was immersed in 25 mL of ultrapure water and shaken at 100 rpm under 25 °C in a refrigerated incubator with a shaker (SHK.109, ETHIK) protected from light. The resultant mixture was removed after 24 h. Analyses were performed in triplicate, and the released silver content in the solution was determined by using FAAS spectroscopy.

#### 2.2.5 Water Contact Angle

The fabric samples were subjected to a water contact angle (WCA) measurement by the sessile drop method. A fabric sample was placed on a flat glass slide, and ultrapure water was dropped onto it. A digital camera was positioned on the side of the sample surface to monitor the drop after 10 s from digital images.^53^ The analysis was performed in duplicate. Images were analyzed by using imaging software (ImageJ), and WCA was measured for each sample.

#### 2.2.6 Tensile Test

The tensile test was carried out using a universal testing machine with two fixed claws adapted for textile testing. The sample fabric had an hourglass shape and a 90 mm^2^ surface area. The test speed was 10 mm.min^–1^, the distance between grips was 40 mm, and the initial load was 2 N to calculate the stress and evaluate Young’s modulus.

#### 2.2.7 Antimicrobial Activity

The agar disk diffusion method was used to evaluate antimicrobial activity on fabric sample surfaces, according to the Clinical & Laboratory Standards Institute.^54^ SA and EC bacteria were used, which were cultivated in Mueller Hinton broth medium at 37 °C for 24 h. Bacterial suspensions were prepared with a saline solution of 0.85% NaCl (w/v) to standardize cell density until reaching 1.0 × 10^8^ CFU·mL^–1^ (colony forming units) on the McFarland scale. Mueller Hinton medium has pH 7.4, which was adjusted to pH 6.0 using aqueous solutions of HCl or NaOH (0.01 mol·L^–1^).^55^ Discs with 6.0 mm diameter fabric samples were added to Petri dishes seeded with the bacteria and incubated for 24 h at 37 °C. The inhibition halos formed were determined in millimeters.

#### 2.2.8 Adhesion and Proliferation Assay of Bacteria

The assay for bacterial adhesion and proliferation on the sample surfaces followed a previously described methodology^56^ with modifications. Bacterial cultures of SA and EC, thawed in a solution of water and glycerol [1:1], were centrifuged for 10 min at 4700 rpm, removing the supernatant and resuspending the precipitate using LB. The resultant solution was incubated for 24 h at 37 °C under agitation at 100 rpm in a shaker incubator. Fabric samples were cut into 6 mm diameter discs (triplicate) and fixed in Teflon holders for seeding at a concentration of 1.0 × 107 cells mL^–1^ in a multiwell plate (48 wells) for 24 h. The bacterial solution was removed from the wells, and the samples were washed with PBS (500 μL at pH 7.4). The fixation of bacteria that adhered to the samples was carried out at room temperature for 45 min by using a 3% (v/v) solution of glutaraldehyde, sodium cacodylate (0.1 mol·L^–1^), and sucrose (0.1 mol·L^–1^). It was subsequently washed with sodium cacodylate buffer (0.1 mol·L^–1^), sucrose (0.1 mol·L^–1^), and dried for 10 min in each solution with a concentration gradient of ethanol/water (35%, 50%, 75%, and 100%), then frozen, and lyophilized for 24 h to be analyzed by SEM.

### 2.3 Statistical Analysis

Statistical analyses of the results from obtaining ordinary one-way ANOVA and multiple comparisons of mean values were evaluated using the Tukey test with 95% confidence.

## 3 Results and Discussion

### 3.1 Synthesis of AgNPs, *k-*CA Film, and *k-*CA/AgNPs Film on Cotton Fabric

**Figure 1:**
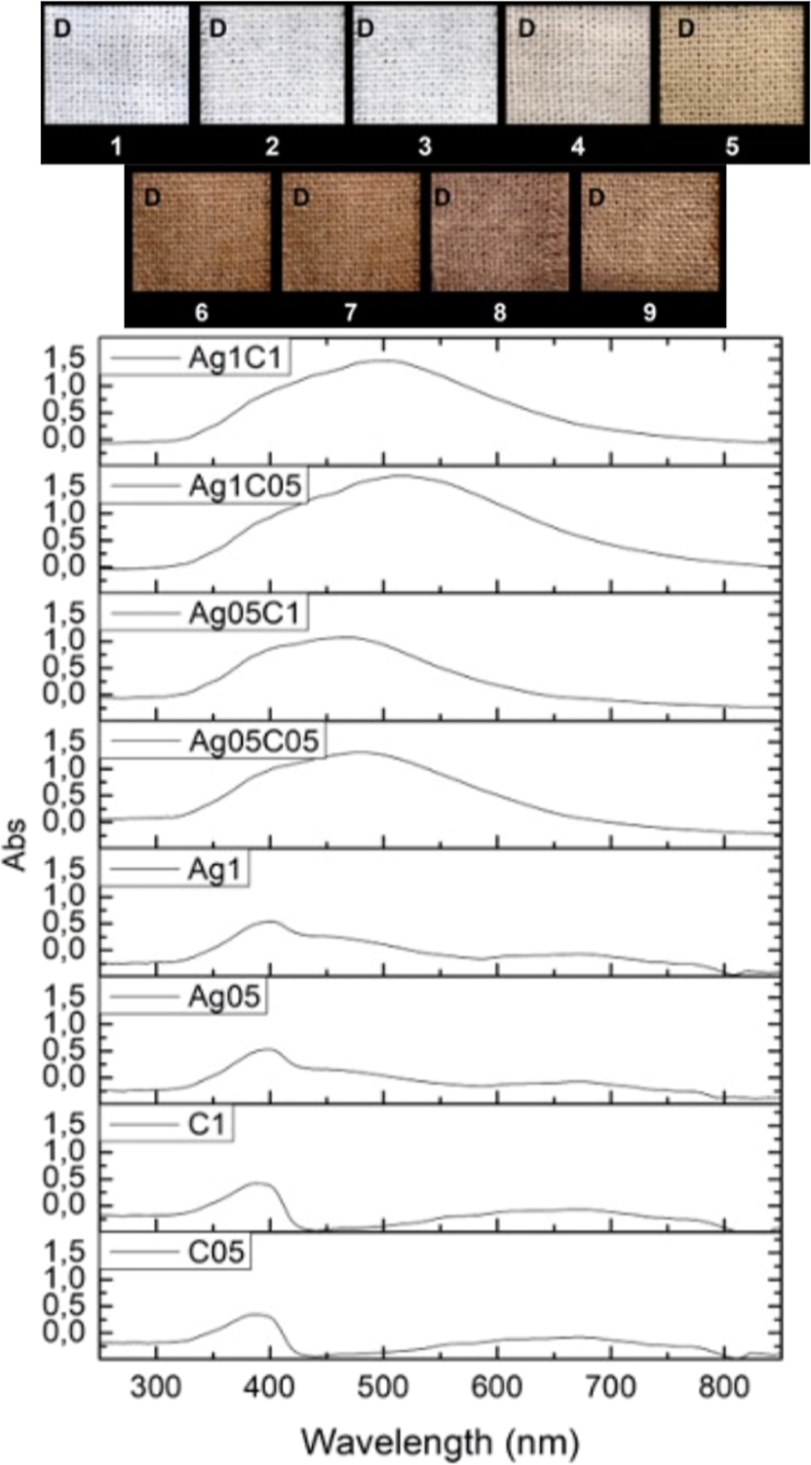
Above: photographic images for the dry cotton samples: (1) bare cotton, (2) C05, (3) C1, (4) Ag05, (5) Ag1, (6) Ag05C05, (7) Ag05C1, (8) Ag1C05, (9) Ag1C1. Below: spectra UV–vis for the dry cotton samples.

Regarding the AgNPs synthesis directly on cotton from the cotton samples immersion in AgNO_3_ solution, the hydroxyl groups of the cellulose chemical structure (cotton) were possibly responsible for the reduction of the silver ions (Ag^+^) to metallic silver (Ag^0^).^57^ This was evident as the initially white fabric surface takes on an orange coloration that is characteristic of the AgNPs formation (compare the images of sample (1) with the images of samples (4) and (5) in Figure 1). The orange color was more intense as the used AgNO_3_ concentration was higher. That indicates a higher level of AgNPs formation. The residual synthesis solution is colorless, which indicates that the AgNPs were only formed on the fabric surface through the reductive action of the cotton fiber’s functional groups.

When the cotton samples were treated with heated *k-*CA solution for both tested concentrations, a light-yellowish hydrogel film was formed on the samples surface after cooling (see samples (2) and (3) in Figure 1). The *k-*CA is a sulfated polysaccharide that forms thermo-reversible hydrogels.^48^ The *k-*CA films were visibly well adhered to the fabric, possibly due to the good interaction between the hydroxyl groups of the *k-*CA polymeric matrices and hydrophilic groups on cellulose.^58,59^

The *k-*CA has been successfully used to produce stable AgNPs from silver nitrate.^24,60^ The negative charges of *k-*CA (sulfated groups) bring the ions Ag^+^ closer to the polymer chain and facilitate their reduction by the hydroxyl groups.^30^ Thus, the AgNPs were obtained by a heated *k-*CA solution using two different concentrations of polysaccharide and AgNO_3_ (silver precursor). The zeta potential and hydrodynamic diameter were obtained by DLS analysis of the AgNPs suspension in *k*-CA (Table 2). The zeta potential values showed that the AgNPs are negative (between ca. −19 and −29 mV), indicating electrostatic stability and spontaneous resistance to aggregation.^25^ The negative charge is explained by the sulfated group in the *k*-CA polymeric chain that stabilizes the AgNPs.^26^ The AgNPs hydrodynamic radius was from ca. 72.1 to 159.2 nm. The radius range obtained was slightly larger than that reported in other studies related to the synthesis of AgNPs in biopolymers that reported radii smaller than 100 nm.^21,61^ However, the particle radius can vary greatly in green synthesis due to the influence of several factors related to the heterogeneity derived from the polymers biological nature.^21^

**Table 2:** Averages Hydrodynamic Diameter and Zeta Potential Obtained by DLS^a^

sample	average hydrodynamic radius (nm)	average zeta potential (ζ) (mV)
A05C05	72.1	–18.9
A05C1	114.7	–20.3
A1C05	159.2	–29.0
A1C1	124.9	–21.3

a Values acquired from duplicates.

After AgNPs synthesis, cotton samples were added to a heated AgNPs suspension to form a *k*-CA/AgNPs composite film on fabric after colling. A more intense orange color on the cotton surface was observed when a AgNPs/*k*-CA composite film was obtained compared to the color intensity obtained from the AgNPs synthesized directly on the cotton. This indicates that more AgNPs were incorporated on the cotton surface, considering the same silver precursor concentration (compare the images of sample (4) with (6) and (7) ones and sample (5) with (8) and (9) ones, in Figure 1).

The AgNPs synthesis was also monitored spectroscopically, and the UV–vis spectra for the coated samples are shown in Figure 1. It was observed that *k-*CA-coated cotton samples (C05 and C1) showed an absorption band before 400 nm due to the *k-*CA hydrogel. The AgNPs-coated cotton samples (Ag05 and Ag1) showed a band at 410 nm due to localized surface plasmon resonance (LSPR) absorbance due to AgNPs presence and a less intense and broadened signal around 450 nm, possibly due to the occurrence of more aggregated and less homogeneous AgNPs.^14,25,37^ Lastly, the *k*-CA/AgNPs-coated cotton samples (Ag05C05, Ag05C1, Ag1C05, and Ag1C1) showed a wide band for all concentrations. The resultant band combined the *k*-CA (before 400 nm) and AgNPs (around 500 nm) absorptions. The AgNPs LSPR band shifts to a longer wavelength for AgNPs embedded into 3D polysaccharides matrices.^21,61,62^ Moreover, the spectra showed that *k*-CA/AgNPs-coated cotton samples had LSPR bands that were more intense than those observed for the AgNPs-coated samples. This confirms that a higher AgNPs amount was inserted on surface fabrics when *k*-CA was used, corroborating the greater intensity of the orange color observed for the *k*-CA/AgNPs-coated cotton samples previously discussed.

Comparing the samples coated with the composite film, it was impossible to notice (based on the orange images in Figure 1) a significant difference in the AgNPs production. Moreover, the absorption spectra showed that the absorbance did not increase when the *k*-CA concentration increased under the same AgNO_3_ concentration (compare the LSPR intensity for the pairs Ag05C05–Ag05C1 and Ag1C05–Ag1C1 in Figure 1). However, the absorbance increased when the AgNO_3_ concentration went from 0.5 to 1.0%, keeping the *k*-CA concentration constant (compare the Ag05C05–Ag1C05 and Ag05C1–Ag1C1 concentrations in Figure 1). This indicates that AgNO_3_ is limiting and must be fully converted into AgNPs, even at the lowest *k*-CA concentration.

### 3.2 Scanning Electron Microscopy and Energy-Dispersive X-ray Spectroscopy

**Figure 2:**
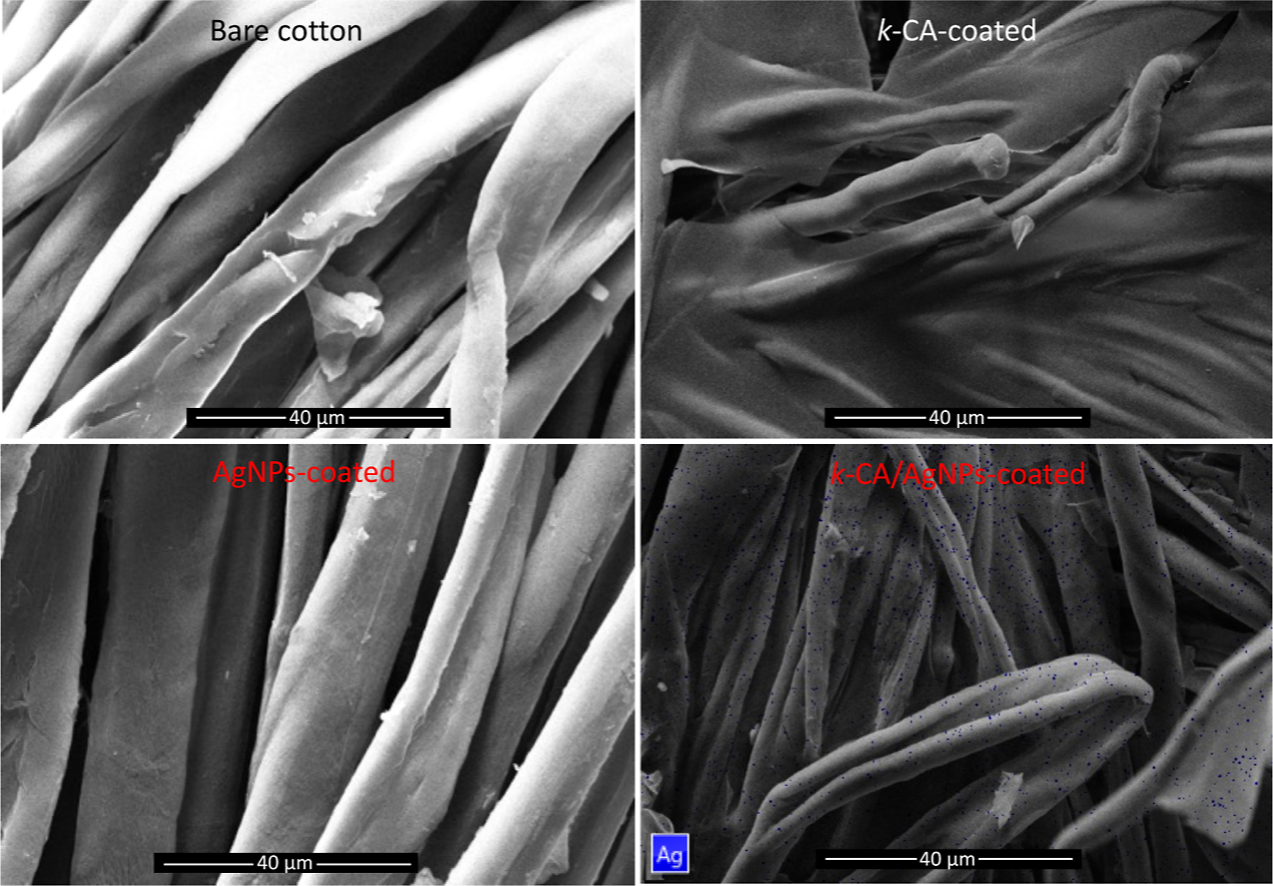
SEM images for different types of cotton samples tested. The blue points in the *k*-CA/AgNPs-coated sample image correspond to the points where the signal for silver was identified via EDS elemental mapping.

The surface morphology was analyzed by using SEM images (Figure 2). The images show a structure of fibers to bare cotton fabric. Similar morphology was also visualized for AgNPs-coated samples. On the other hand, a smooth and discontinuous film was clearly obtained on the fiber surfaces for the *k*-CA-coated and *k*-CA/AgNPs-coated samples. This corroborates the fact that a *k*-CA hydrogel film was produced on the cotton. The coating or film morphology characteristics did not vary significantly with respect to the variation in the concentration of *k-*CA or the silver precursor used in the synthesis. The AgNPs presence could not be directly visualized through SEM images.

**Figure 3:**
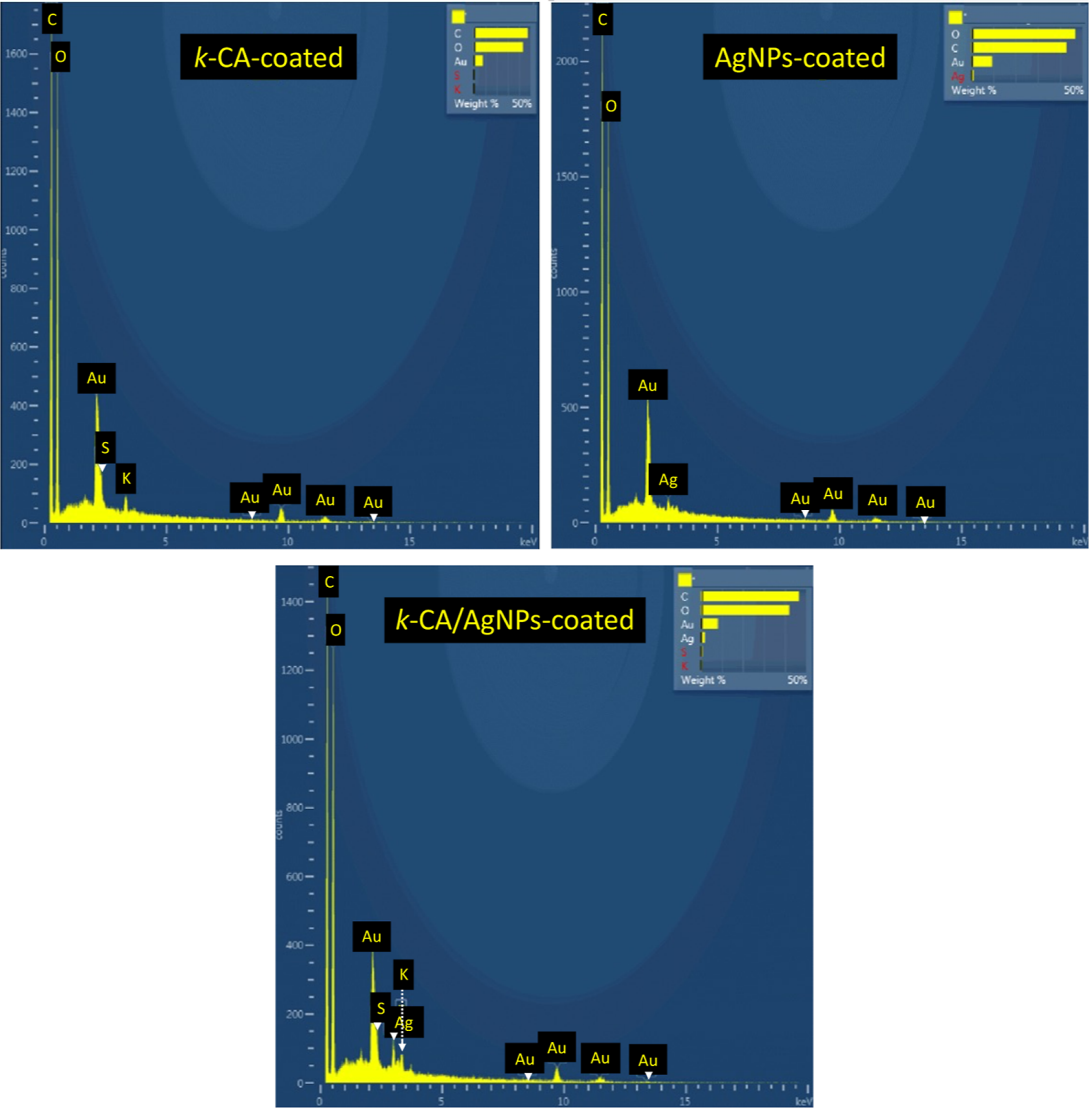
EDS spectra for different coated cotton samples.

The surface samples were evaluated by EDS, and Figure 3 shows the spectra of some samples. Gold, carbon, and oxygen peaks were observed in all of the samples. Gold signals are due to sample preparation (gold metallization). The carbon and oxygen peaks, on the other hand, come from the cotton and *k-*CA polymeric chains. The sulfur peak was observed for the *k*-CA and *k*-CA/AgNPs-coated samples and confirms the presence of the sulfated polysaccharide.^48^ The potassium signal for the same samples comes from counterions in the *k-*CA matrix or *k-*CA impurities. Lastly, the Ag peaks confirm the presence of silver metal in nanoparticulate or small agglomerate forms. Mapping the distribution of silver on the surface of a *k*-CA/AgNPs-coated sample (Figure 2) shows that the metal is distributed where there is the hydrogel film, indicating that the AgNPs are embedded in the *k*-CA matrix. EDS spectrum for bare cotton was very similar to that obtained for k-CA-coated samples except for the nonoccurrence of S and K signals (characteristic of *k*-CA). It was not possible to observe a relevant influence on the EDS spectrum for the different concentrations of Ag and *k*-CA used.

### 3.3 Silver Incorporation and Release

The sample silver amount (in AgNPs form) was obtained from a sample acid digestion. Then, the free Ag^+^ ions were dosed by FAAS after digestion by comparing it with standard Ag^+^ solutions.^12^ The results are expressed in Table 3. It was found that the *k*-CA/AgNPs-coated fabrics had greater amounts of AgNPs (average quantity varied between 0.45 and 0.75 mg) compared with AgNPs-coated samples (average quantity varied between 0.27 and 0.33 mg). This corroborates what was previously discussed about the absorbance spectra for the samples. Statistical analyses (Figure S1) showed that the *k*-CA/AgNPs-coated samples obtained at a higher silver precursor concentration (Ag1C05 and Ag1C1) incorporated significantly more silver onto the fabric surface than the AgNPs-coated samples (Ag05 and Ag1). The AgNPs amount that comes from the most loaded *k*-CA/AgNPs-coated sample (Ag1C05) was ca. 2.3 times higher than that of the most loaded sample that did not use the polysaccharide (Ag1). The greater loading of AgNPs in *k*-CA-coated samples is that AgNPs are produced throughout the polysaccharide film volume, while the loading of AgNPs directly onto cotton samples is limited to its surface area (see Figure S2). Furthermore, it was proven that using the smallest amount of *k*-CA in the synthesis (0.5% *k*-CA) is enough to reduce Ag^+^ ions even at the highest concentration tested (1% AgNO_3_). This can be stated because the amounts of AgNPs from Ag1C05 and Ag1C1 (or Ag05C05 and Ag05C1) are statistically equal (Figure S1).

**Table 3:** Silver Concentrations after 12 h in the Release Assay for the Samples

sample	average amount of incorporated Ag (mg)^a^	normalized average concentration of released Ag (mg·L^–1^cm^–2^)^a^
C05	0.0 ± 0.0	0.00 ± 0.00
C1	0.0 ± 0.0	0.00 ± 0.00
Ag05	0.27 ± 0.06	0.23 ± 0.03
Ag1	0.33 ± 0.05	0.22 ± 0.03
Ag05C05	0.55 ± 0.02	0.25 ± 0.01
Ag05C1	0.45 ± 0.04	0.24 ± 0.00
Ag1C05	0.75 ± 0.08	0.38 ± 0.05
Ag1C1	0.66 ± 0.03	0.32 ± 0.02

a Average obtained from triplicates.

Afterward, the cotton samples were immersed in water and slowly agitated for 24 h to assess the released silver. After this, Ag^+^ present in the resulting liquid phase was quantified by FAAS. Table 3 shows the released silver ion concentrations, normalized by cm^2^, which were obtained from the fabric samples.

It was observed that the amount of silver released by the samples did not have very discrepant levels. Statistical analyses showed that the released silver concentration from sample Ag1C05 differed significantly from all others except sample Ag1C1 (see Figure S3). Then, the *k*-CA/AgNPs-coated samples obtained using a 1% silver precursor stood out, having achieved the highest levels of released silver (0.38 and 0.32 mg·L^–1^cm^–2^, respectively). This demonstrates that the polysaccharide does not act as a barrier to the diffusion of silver from the samples; conversely, the hydrogel created a path for the facilitated exit of Ag^+^ ions. The released silver concentrations by the samples should not cause any type of toxicity in humans; in addition, silver does not trigger any irritation to the skin.^63^ Using any *k*-CA/AgNPs samples incorporates an extra advantage because their higher AgNPs amount (regarding the AgNPs-coated sample, as previously discussed) could sustain the silver release for a longer time.

### 3.4 WCA and Mechanical Test

The WCA is used to assess the samples surface wettability.^64^ The wettability is obtained by the angle measurement based on droplet behavior on the fabric substrates, as shown in Table 4 inset. If the WCA is greater than 90°, the sample is considered hydrophobic; if the WCA is less than 90°, the sample is hydrophilic.^56,65^

**Table 4:** WCA for Samples^a^

sample	WCA (deg)^b^
C05	104 ± 1
C1	104 ± 2
Ag05	109 ± 3
Ag1	106 ± 2
Ag05C05	107 ± 3
Ag1C05	103 ± 5
Ag05C1	112 ± 4
Ag1C1	105 ± 4

a Statistical test shows no significant variation among values.

b Average obtained from triplicates.

Table 4 shows that all textile samples had hydrophobic behavior (WCA ≥ 90°). Thus, the AgNPs and/or *k*-CA presence did not show a significant variation in cotton wettability. Wettability is important for dressings applications, as they can collaborate with wound healing, for example.^13,47^ The higher hydrophobicity may also contribute to less microorganism adhesion.

**Figure 4:**
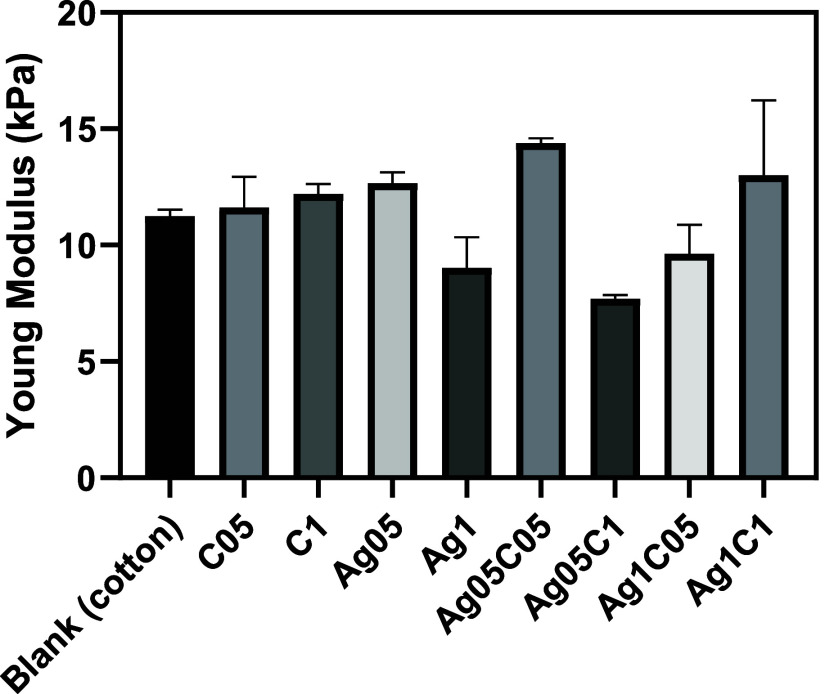
Young’ moduli were obtained from a tensile test for the samples. Statistical analysis shows no significant variation between average values for the coated samples and bare cotton (blank).

Fabric samples were also subjected to tensile tests to evaluate possible changes in the cotton’s mechanical properties after surface modifications. Processes that involve heating or application of chemical reagents can compromise the fibers strength. Figure 4 shows the average Young’s moduli obtained for the samples. No significant difference was observed between Young’s moduli for the coated samples (*k*-CA, AgNPs, and *k*-CA/AgNPs) and bare cotton. This indicates that the conditions for obtaining the coatings were not sufficient to cause any change in the resistance of the cotton fabric.

### 3.5 Antimicrobial and Anti-Adhesive Activities

**Figure 5:**
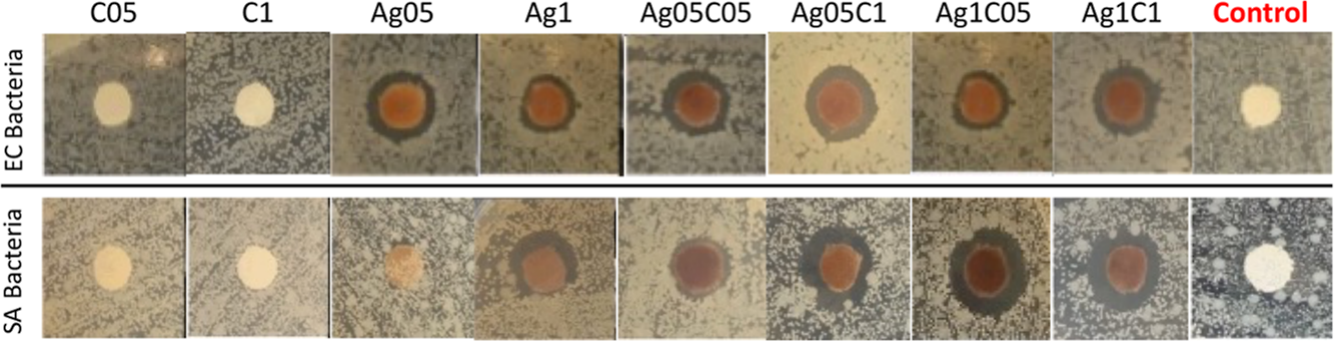
Inhibition halo against EC and SA bacteria after 24 h. The “control” is cotton without any surface treatment.

The sample antibacterial behavior was evaluated by analyzing the sensitivity of the bacterial growth using the agar disk diffusion technique. Figure 5 shows that both the bacteria (SA and EC) grew homogeneously in the presence of all samples without AgNPs and control (bare cotton sample), indicating a lack of antimicrobial activity.^8^

All samples containing AgNPs, except sample Ag05 against SA, showed an inhibition halo against both Gram-positive and Gram-negative bacteria classes, indicating the bactericidal action of the samples due to the AgNPs oxidation and releasing silver ions.^6,34^ Thus, the samples effectively prevented the bacteria growth on the sample and in the halo region surrounding the sample due to the diffusion of Ag^+^ ions.

Table 5 shows the inhibition halos obtained for the samples that showed better bactericidal performance against both bacteria tested. In general, these samples showed more pronounced bactericidal behavior (greater inhibition halo) for SA. The bactericidal mechanism of the tested coatings must occur due to the released Ag^+^ action. The AgNPs release must not occur because they are adsorbed in the *k*-CA matrix or cellulose (cotton). Therefore, Gram-positive bacteria (such as SA) should be more susceptible to the Ag^+^ ions, as they have a thick cellular envelope formed on the outside by a thick layer of peptidoglycan, which is negatively charged. This should more easily trap Ag^+^ ions, which leads to cell wall disruption and cell death.^66,67^

**Table 5:** Inhibition Halo Diameter Measurements after 24 h for the AgNPs-Coated and *k*-CA/AgNPs-Coated Cotton Samples

	inhibition halo diameter (mm)^a^	
sample	E. coli	S. Aureus
Ag1	6.4 ± 0.5	7.6 ± 0.6
Ag05C1	6.8 ± 0.2	9.3 ± 0.3
Ag1C05	6.2 ± 0.5	7.9 ± 0.1
Ag1C1	6.9 ± 0.2	9.5 ± 0.9

a Average obtained from triplicates.

The bactericidal action against EC bacteria had no significant difference among the samples in Table 3. However, regarding SA bacteria, Ag1C05 and Ag1C1 stood out, showing a significant difference with respect to Ag1. The better bactericidal performance of *k*-CA/AgNPs-coated samples with respect to AgNPs-coated ones must be directly related to their higher amounts of Ag^+^ released in aqueous media (as previously discussed in the silver release test).

**Figure 6:**
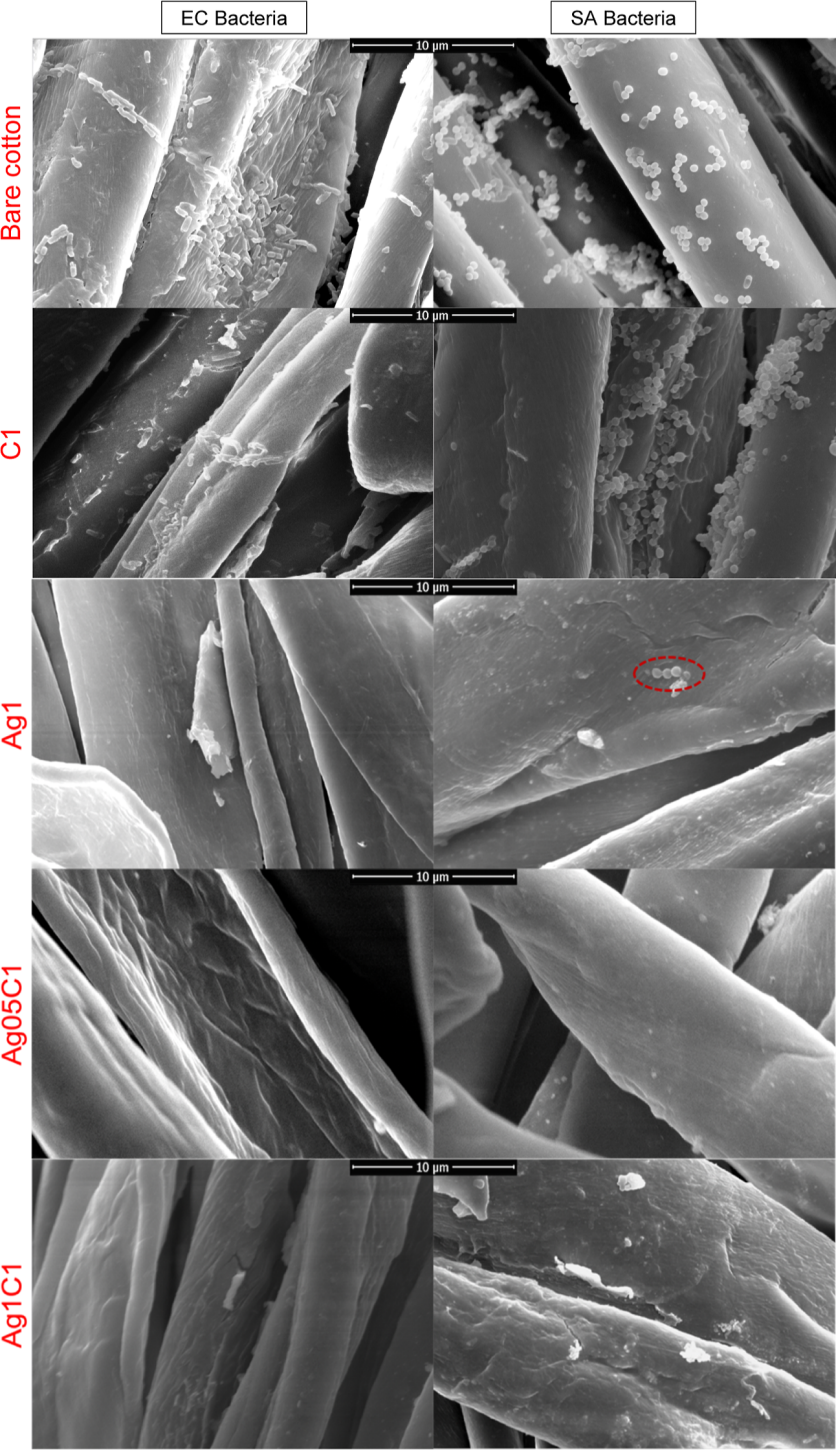
SEM images for the samples after the antiadhesive assay against EC (rod shape) and SA (spherical shape) bacteria.

The antiadhesive assay has corroborated what was observed at the halo inhibition test. The SEM images in Figure 6 show that the EC (rod shape) and SA (spherical shape) bacteria adhered to and proliferated on the surface of the fibers for the *k*-CA-coated and bare cotton samples. On the other hand, the bacteria have not been observed on the surfaces for both AgNPs-coated and *k*-CA/AgNPs-coated samples. It was verified that very few SA cells occur on Ag1 only (see the dashed and red-lined oval form in Figure 6). The results have proved that these materials showed antiadhesive properties for the tested bacteria.^65^

## 4 Conclusions

Here, it was showed the development of a novel bactericidal coating based on *k*-CA/AgNPs hydrogel for cotton fabric. EDS, SEM, and spectrophotometric analyses confirmed the composite obtention on cotton surfaces in a film form. The bactericidal performance of *k*-CA/AgNPs-coating was compared with AgNPs-coating. SEM images showed that all coated samples were antiadhesive against EC and SA, although small traces of SA cells were found on the Ag1 sample. Overall, in vitro diffusion bactericidal tests showed that all coated samples tested did not allow the growth of SA and EC, and an inhibition halo was observed, except for the Ag05 sample against SA. Cotton fabrics covered with both finishes based on AgNPs obtained from 1% AgNO_3_ (with and without *k*-CA) had similar bactericidal activity against EC, resulting in 6.2 and 6.9 mm halos for the Ag1C1 and Ag1, respectively. However, the *k*-CA/AgNPs-coated samples had significantly better performance with respect to SA, and the highest halo was reached (9.5 mm for Ag1C1 against 7.6 mm for the Ag1). This was due to the greater capacity to release Ag^+^ ions observed for samples coated with the composite (0.32 mg·L^–1^ cm^2^ for Ag1C1 against 0.22 mg·L^–1^ cm^2^ for the Ag1). Analysis of the halos also showed that there is a more intense bactericidal action of all samples against SA (more sensitive to the Ag^+^ ions). Furthermore, it was shown that the Ag1C1 sample incorporated two times more AgNPs than Ag1 on the cotton fabric surface. This shows that the *k*-CA/AgNPs-coated sample, in addition to having similar bactericidal performance against EC and better against SA, is also more advantageous as it is able to sustain this functionality for longer. Thus, this should be a great coating condition for cotton for medical applications where a bactericidal surface is required, such as wound healing or medical clothing.

## Supporting Information Available

Statistical analyses of the data obtained from FAAS for the quantification of silver incorporated on the fabric and silver released and a scheme of silver incorporation and release from the cotton samples (PDF)

## References

[ref1] Cheng H.; Gu B.; Pennefather M. P.; Nguyen T. X.; Phan-Thien N.; Duong H. M. Cotton Aerogels and Cotton-Cellulose Aerogels from Environmental Waste for Oil Spillage Cleanup. Mater. Des. 2017, 130, 452–458. 10.1016/j.matdes.2017.05.082.

[ref2] Souza P. R.; de Oliveira A. C.; Vilsinski B. H.; Kipper M. J.; Martins A. F. Polysaccharide-Based Materials Created by Physical Processes: From Preparation to Biomedical Applications. Pharmaceutics 2021, 13 (5), 621 10.3390/pharmaceutics13050621. 33925380 PMC8146878

[ref3] Carapeto A. P.; Ferraria A. M.; Botelho do Rego A. M. Silver Nanoparticles on Cellulose Surfaces: Quantitative Measurements. Nanomaterials 2019, 9 (5), 780 10.3390/nano9050780. 31121849 PMC6566189

[ref4] Xu Q.; Li R.; Shen L.; Xu W.; Wang J.; Jiang Q.; Zhang L.; Fu F.; Fu Y.; Liu X. Enhancing the Surface Affinity with Silver Nano-Particles for Antibacterial Cotton Fabric by Coating Carboxymethyl Chitosan and L-Cysteine. Appl. Surf. Sci. 2019, 497, 143673 10.1016/j.apsusc.2019.143673.

[ref5] Maghimaa M.; Alharbi S. A. Green Synthesis of Silver Nanoparticles from Curcuma Longa L. and Coating on the Cotton Fabrics for Antimicrobial Applications and Wound Healing Activity. J. Photochem. Photobiol., B 2020, 204 (December 2019), 111806 10.1016/j.jphotobiol.2020.111806. 32044619

[ref6] Zepon K. M.; Marques M. S.; da Silva Paula M. M.; Morisso F. D. P.; Kanis L. A. Facile, Green and Scalable Method to Produce Carrageenan-Based Hydrogel Containing in Situ Synthesized AgNPs for Application as Wound Dressing. Int. J. Biol. Macromol. 2018, 113, 51–58. 10.1016/j.ijbiomac.2018.02.096. 29471089

[ref7] Zhang S.; Kai C.; Liu B.; Zhang S.; Wei W.; Xu X.; Zhou Z. Facile Fabrication of Cellulose Membrane Containing Polyiodides and Its Antibacterial Properties. Appl. Surf. Sci. 2020, 500, 144046 10.1016/j.apsusc.2019.144046.

[ref8] Zia T.; Usman M.; Sabir A.; Shafiq M.; Khan R. U. Development of Inter-Polymeric Complex of Anionic Polysaccharides, Alginate/k-Carrageenan Bio-Platform for Burn Dressing. Int. J. Biol. Macromol. 2020, 157, 83–95. 10.1016/j.ijbiomac.2020.04.157. 32335110

[ref9] Islam M. T.; Al Mamun M. A.; Hasan M. T.; Shahariar H. Scalable Coating Process of AgNPs-Silicone on Cotton Fabric for Developing Hydrophobic and Antimicrobial Properties. J. Coat. Technol. Res. 2021, 18 (3), 887–898. 10.1007/s11998-020-00451-z.

[ref10] Said M. M.; Rehan M.; El-Sheikh S. M.; Zahran M. K.; Abdel-Aziz M. S.; Bechelany M.; Barhoum A. Multifunctional Hydroxyapatite/Silver Nanoparticles/Cotton Gauze for Antimicrobial and Biomedical Applications. Nanomaterials 2021, 11 (2), 429 10.3390/nano11020429. 33567743 PMC7915402

[ref11] Kouhi M.; Prabhakaran M. P.; Ramakrishna S. Edible Polymers: An Insight into Its Application in Food, Biomedicine and Cosmetics. Trends Food Sci. Technol. 2020, 103 (May), 248–263. 10.1016/j.tifs.2020.05.025.

[ref12] Marbun M. Y. E.; Helmiyati H. Hydrogel Nanocomposites SA-PAA Modified by Silver Nanoparticle (AgNPs) for Antibacterial Application on Cotton Fabrics. AIP Conference Proceedings; American Institute of Physics Inc., 2020; Vol. 2242.

[ref13] Tao G.; Cai R.; Wang Y.; Liu L.; Zuo H.; Zhao P.; Umar A.; Mao C.; Xia Q.; He H. Bioinspired Design of AgNPs Embedded Silk Sericin-Based Sponges for Efficiently Combating Bacteria and Promoting Wound Healing. Mater. Des. 2019, 180, 107940 10.1016/j.matdes.2019.107940.

[ref14] Štular D.; Savio E.; Simončič B.; Šobak M.; Jerman I.; Poljanšek I.; Ferri A.; Tomšič B. Multifunctional Antibacterial and Ultraviolet Protective Cotton Cellulose Developed by in Situ Biosynthesis of Silver Nanoparticles into a Polysiloxane Matrix Mediated by Sumac Leaf Extract. Appl. Surf. Sci. 2021, 563, 150361 10.1016/j.apsusc.2021.150361.

[ref15] Yildiz A.; Vatansever Bayramol D.; Atav R.; Ağirgan A. Ö.; Aydin Kurç M.; Ergünay U.; Mayer C.; Hadimani R. L. Synthesis and Characterization of Fe3O4@Cs@Ag Nanocomposite and Its Use in the Production of Magnetic and Antibacterial Nanofibrous Membranes. Appl. Surf. Sci. 2020, 521, 146332 10.1016/j.apsusc.2020.146332.

[ref16] Jiang S.; Tang C.; Gong Z.; Zhang Z.; Wang D.; Fan M. Facile Preparation of Chitosan Coated Silver Nanoparticles Embedded Cotton Fabric for Point-of-Use Water Disinfection. Mater. Lett. 2020, 277, 128256 10.1016/j.matlet.2020.128256.

[ref17] Elsupikhe R. F.; Shameli K.; Ahmad M. B.; Ibrahim N. A.; Zainudin N. Green Sonochemical Synthesis of Silver Nanoparticles at Varying Concentrations of κ-Carrageenan. Nanoscale Res. Lett. 2015, 10 (1), 302 10.1186/s11671-015-0916-1. 26220106 PMC4523502

[ref18] Zhang X. F.; Liu Z. G.; Shen W.; Gurunathan S. Silver Nanoparticles: Synthesis, Characterization, Properties, Applications, and Therapeutic Approaches. Int. J. Mol. Sci. 2016, 17 (9), 1534 10.3390/ijms17091534. 27649147 PMC5037809

[ref19] Öztürk İ.; Beğiç N.; Bener M.; Apak R. Antioxidant Capacity Measurement Based on κ-Carrageenan Stabilized and Capped Silver Nanoparticles Using Green Nanotechnology. J. Mol. Struct. 2021, 1242, 130846 10.1016/j.molstruc.2021.130846.

[ref20] Lobregas M. O. S.; Bantang J. P. O.; Camacho D. H. Carrageenan-Stabilized Silver Nanoparticle Gel Probe Kit for Colorimetric Sensing of Mercury (II) Using Digital Image Analysis. Sens Biosensing Res. 2019, 26, 100303 10.1016/j.sbsr.2019.100303.

[ref21] Noralian Z.; Gashti M. P.; Moghaddam M. R.; Tayyeb H.; Erfanian I. Ultrasonically Developed Silver/Iota-Carrageenan/Cotton Bionanocomposite as an Efficient Material for Biomedical Applications. Int. J. Biol. Macromol. 2021, 180, 439–457. 10.1016/j.ijbiomac.2021.02.204. 33705835

[ref22] Eswaran S. G.; Narayan H.; Vasimalai N.; Vasimalai N. Reductive Photocatalytic Degradation of Toxic Aniline Blue Dye Using Green Synthesized Banyan Aerial Root Extract Derived Silver Nanoparticles. Biocatal. Agric. Biotechnol. 2021, 36, 102140 10.1016/j.bcab.2021.102140.

[ref23] Spagnoletti F. N.; Kronberg F.; Spedalieri C.; Munarriz E.; Giacometti R. Protein Corona on Biogenic Silver Nanoparticles Provides Higher Stability and Protects Cells from Toxicity in Comparison to Chemical Nanoparticles. J. Environ. Manage. 2021, 297 (July), 113434 10.1016/j.jenvman.2021.113434. 34400389

[ref24] Wan H.; Li C.; Mahmud S.; Liu H. Kappa Carrageenan Reduced-Stabilized Colloidal Silver Nanoparticles for the Degradation of Toxic Azo Compounds. Colloids Surf., A 2021, 616, 126325 10.1016/j.colsurfa.2021.126325.

[ref25] Roy S.; Shankar S.; Rhim J. W. Melanin-Mediated Synthesis of Silver Nanoparticle and Its Use for the Preparation of Carrageenan-Based Antibacterial Films. Food Hydrocolloids 2019, 88, 237–246. 10.1016/j.foodhyd.2018.10.013.

[ref26] Pandey S.; Do J. Y.; Kim J.; Kang M. Fast and Highly Efficient Catalytic Degradation of Dyes Using κ-Carrageenan Stabilized Silver Nanoparticles Nanocatalyst. Carbohydr. Polym. 2020, 230, 115597 10.1016/j.carbpol.2019.115597. 31887912

[ref27] Singh A.; Gaud B.; Jaybhaye S. Optimization of Synthesis Parameters of Silver Nanoparticles and Its Antimicrobial Activity. Mater. Sci. Energy Technol. 2020, 3, 232–236. 10.1016/j.mset.2019.08.004.

[ref28] Yegappan R.; Selvaprithiviraj V.; Amirthalingam S.; Jayakumar R. Carrageenan Based Hydrogels for Drug Delivery, Tissue Engineering and Wound Healing. In Carbohydrate Polymers; Elsevier Ltd October, 2018; Vol. 15, pp 385–400. 10.1016/j.carbpol.2018.06.086 30093014

[ref29] El-Shishtawy R. M.; Asiri A. M.; Abdelwahed N. A. M.; Al-Otaibi M. M. In Situ Production of Silver Nanoparticle on Cotton Fabric and Its Antimicrobial Evaluation. Cellulose 2011, 18 (1), 75–82. 10.1007/s10570-010-9455-1.

[ref30] Wang Y.; Dong X.; Zhao L.; Xue Y.; Zhao X.; Li Q.; Xia Y. Facile and Green Fabrication of Carrageenan-Silver Nanoparticles for Colorimetric Determination of Cu2+ and S2–. Nanomaterials 2020, 10 (1), 83 10.3390/nano10010083. 31906386 PMC7023203

[ref31] Abutaha N.; Hezam A.; Almekhlafi F. A.; Saeed A. M. N.; Namratha K.; Byrappa K. Rational Design of Ag-ZnO-Fe3O4 Nanocomposite with Promising Antimicrobial Activity under LED Light Illumination. Appl. Surf. Sci. 2020, 527, 146893 10.1016/j.apsusc.2020.146893.

[ref32] Nogueira S. S.; de Araujo-Nobre A. R.; Mafud A. C.; Guimarães M. A.; Alves M. M. M.; Plácido A.; Carvalho F. A. A.; Arcanjo D. D. R.; Mascarenhas Y.; Costa F. G.; Albuquerque P.; Eaton P.; de Souza de Almeida Leite J. R.; da Silva D. A.; Cardoso V. S. Silver Nanoparticle Stabilized by Hydrolyzed Collagen and Natural Polymers: Synthesis, Characterization and Antibacterial-Antifungal Evaluation. Int. J. Biol. Macromol. 2019, 135, 808–814. 10.1016/j.ijbiomac.2019.05.214. 31158421

[ref33] Elsayed H.; Hasanin M.; Rehan M. Enhancement of Multifunctional Properties of Leather Surface Decorated with Silver Nanoparticles (Ag NPs). J. Mol. Struct. 2021, 1234, 130130 10.1016/j.molstruc.2021.130130.

[ref34] Bonilla-Gameros L.; Chevallier P.; Sarkissian A.; Mantovani D. Silver-Based Antibacterial Strategies for Healthcare-Associated Infections: Processes, Challenges, and Regulations. An Integrated Review. Nanomedicine 2020, 24, 102142 10.1016/j.nano.2019.102142. 31843661

[ref35] Souza P. R.; Vilsinski B. H.; de Oliveira A. C.; Berton S. B. R.; Nunes C. S.; Kipper M. J.; Schrekker H. S.; Martins A. F.; Muniz E. C. Chitosan/Heparin Blends in Ionic Liquid Produce Polyelectrolyte Complexes That Quickly Adsorb Citrate-Capped Silver Nanoparticles, Forming Bactericidal Composites. J. Mol. Liq. 2021, 330, 115548 10.1016/j.molliq.2021.115548.

[ref36] Kędziora A.; Speruda M.; Krzyżewska E.; Rybka J.; Łukowiak A.; Bugla-Płoskońska G. Similarities and Differences between Silver Ions and Silver in Nanoforms as Antibacterial Agents. Int. J. Mol. Sci. 2018, 19, 444 10.3390/ijms19020444. 29393866 PMC5855666

[ref37] Rajput D.; Paul S.; Gupta A. Green Synthesis of Silver Nanoparticles Using Waste Tea Leaves. Adv. NanoBiomed Res. 2020, 3 (1), 1–14. 10.21467/anr.3.1.1-14.

[ref38] Nilavukkarasi M.; Vijayakumar S.; Prathip Kumar S. Biological Synthesis and Characterization of Silver Nanoparticles with Capparis Zeylanica L. Leaf Extract for Potent Antimicrobial and Anti Proliferation Efficiency. Mater. Sci. Energy Technol. 2020, 3, 371–376. 10.1016/j.mset.2020.02.008.

[ref39] Ahmed T.; Ogulata R. T. A Review on Silver Nanoparticles -Green Synthesis, Antimicrobial Action and Application in Textiles. J. Nat. Fibers 2022, 19 (14), 8463–8484. 10.1080/15440478.2021.1964135.

[ref40] Lin J.; Chen X.; Chen C.; Hu J.; Zhou C.; Cai X.; Wang W.; Zheng C.; Zhang P.; Cheng J.; Guo Z.; Liu H. Durably Antibacterial and Bacterially Antiadhesive Cotton Fabrics Coated by Cationic Fluorinated Polymers. ACS Appl. Mater. Interfaces 2018, 10 (7), 6124–6136. 10.1021/acsami.7b16235. 29356496

[ref41] Liu Y.; Li F.; Guo Z.; Xiao Y.; Zhang Y.; Sun X.; Zhe T.; Cao Y.; Wang L.; Lu Q.; Wang J. Silver Nanoparticle-Embedded Hydrogel as a Photothermal Platform for Combating Bacterial Infections. Chem. Eng. J. 2020, 382, 122990 10.1016/j.cej.2019.122990.

[ref42] Travan A.; Pelillo C.; Donati I.; Marsich E.; Benincasa M.; Scarpa T.; Semeraro S.; Turco G.; Gennaro R.; Paoletti S. Non-Cytotoxic Silver Nanoparticle-Polysaccharide Nanocomposites with Antimicrobial Activity. Biomacromolecules 2009, 10 (6), 1429–1435. 10.1021/bm900039x. 19405545

[ref43] El-Naggar M. E.; Othman S. I.; Allam A. A.; Morsy O. M. Synthesis, Drying Process and Medical Application of Polysaccharide-Based Aerogels. Int. J. Biol. Macromol. 2020, 145, 1115–1128. 10.1016/j.ijbiomac.2019.10.037. 31678101

[ref44] Rahimi M.; Noruzi E. B.; Sheykhsaran E.; Ebadi B.; Kariminezhad Z.; Molaparast M.; Mehrabani M. G.; Mehramouz B.; Yousefi M.; Ahmadi R.; Yousefi B.; Ganbarov K.; Kamounah F. S.; Shafiei-Irannejad V.; Kafil H. S. Carbohydrate Polymer-Based Silver Nanocomposites: Recent Progress in the Antimicrobial Wound Dressings. Carbohydr. Polym. 2020, 231, 115696 10.1016/j.carbpol.2019.115696. 31888835

[ref45] Vijayakumar S.; Saravanakumar K.; Malaikozhundan B.; Divya M.; Vaseeharan B.; Durán-Lara E. F.; Wang M. H. Biopolymer K-Carrageenan Wrapped ZnO Nanoparticles as Drug Delivery Vehicles for Anti MRSA Therapy. Int. J. Biol. Macromol. 2020, 144, 9–18. 10.1016/j.ijbiomac.2019.12.030. 31821826

[ref46] de Lima Barizão C.; Crepaldi M. I.; Junior O. d. O. S.; de Oliveira A. C.; Martins A. F.; Garcia P. S.; Bonafé E. G. Biodegradable Films Based on Commercial κ-Carrageenan and Cassava Starch to Achieve Low Production Costs. Int. J. Biol. Macromol. 2020, 165, 582–590. 10.1016/j.ijbiomac.2020.09.150. 32991902

[ref47] Muthulakshmi L.; Pavithra U.; Sivaranjani V.; Balasubramanian N.; Sakthivel K. M.; Pruncu C. I. A Novel Ag/Carrageenan–Gelatin Hybrid Hydrogel Nanocomposite and Its Biological Applications: Preparation and Characterization. J. Mech. Behav. Biomed. Mater. 2021, 115, 104257 10.1016/j.jmbbm.2020.104257. 33333481

[ref48] Goel A.; Meher M. K.; Gupta P.; Gulati K.; Pruthi V.; Poluri K. M. Microwave Assisted κ-Carrageenan Capped Silver Nanocomposites for Eradication of Bacterial Biofilms. Carbohydr. Polym. 2019, 206, 854–862. 10.1016/j.carbpol.2018.11.033. 30553393

[ref49] Mokhtari H.; Tavakoli S.; Safarpour F.; Kharaziha M.; Bakhsheshi-Rad H. R.; Ramakrishna S.; Berto F. Recent Advances in Chemically-Modified and Hybrid Carrageenan-Based Platforms for Drug Delivery, Wound Healing, and Tissue Engineering. Polymers 2021, 13, 1744 MDPI AG June 1 10.3390/polym13111744. 34073518 PMC8198092

[ref50] Jaiswal L.; Shankar S.; Rhim J. W. Carrageenan-Based Functional Hydrogel Film Reinforced with Sulfur Nanoparticles and Grapefruit Seed Extract for Wound Healing Application. Carbohydr. Polym. 2019, 224, 115191 10.1016/j.carbpol.2019.115191. 31472875

[ref51] de Almeida D. A.; de Oliveira A. C.; Klein R. S.; Bonafé E. G.; Kipper M. J.; Martins A. F.; Monteiro J. P. κ-Carrageenan-Capped Core–Shell Gold@silver Nanoparticles: Optical Device for Hydrogen Peroxide Detection. Nano-Struct. Nano-Objects 2022, 30, 100861 10.1016/j.nanoso.2022.100861.

[ref52] Berton S. B. R.; de Jesus G. A. M.; Sabino R. M.; Monteiro J. P.; Venter S. A. S.; Bruschi M. L.; Popat K. C.; Matsushita M.; Martins A. F.; Bonafé E. G. Properties of a Commercial κ-Carrageenan Food Ingredient and Its Durable Superabsorbent Hydrogels. Carbohydr. Res. 2020, 487, 107883 10.1016/j.carres.2019.107883. 31809910

[ref53] Sandanuwan T.; Hendeniya N.; Amarasinghe D. A. S.; Attygalle D.; Weragoda S. The Effect of Atmospheric Pressure Plasma Treatment on Wetting and Absorbance Properties of Cotton Fabric. In Materials Today: Proceedings; Elsevier Ltd, 2021; Vol. 45, pp 5065–5068.

[ref54] Clinical and Laboratory Standards Institute. Performance Standards for Antimicrobial Disk Susceptibility Tests: Approved Standard, 11th ed.; CLSI, M02-A11, 2012; Vol. 32.

[ref55] Martins J. G.; Facchi D. P.; Berton S. B. R.; Nunes C. S.; Matsushita M.; Bonafé E. G.; Popat K. C.; Almeida V. C.; Kipper M. J.; Martins A. F. Removal of Cu(II) from Aqueous Solutions Imparted by a Pectin-Based Film: Cytocompatibility, Antimicrobial, Kinetic, and Equilibrium Studies. Int. J. Biol. Macromol. 2020, 152, 77–89. 10.1016/j.ijbiomac.2020.02.220. 32092423

[ref56] Plath A. M. S.; Facchi S. P.; Souza P. R.; Sabino R. M.; Corradini E.; Muniz E. C.; Popat K. C.; Filho L. C.; Kipper M. J.; Martins A. F. Zein Supports Scaffolding Capacity toward Mammalian Cells and Bactericidal and Antiadhesive Properties on Poly(ε-Caprolactone)/Zein Electrospun Fibers. Mater. Today Chem. 2021, 20, 100465 10.1016/j.mtchem.2021.100465.

[ref57] Martins A. F.; Follmann H. D. M.; Monteiro J. P.; Bonafé E. G.; Nocchi S.; Silva C. T. P.; Nakamura C. V.; Girotto E. M.; Rubira A. F.; Muniz E. C. Polyelectrolyte Complex Containing Silver Nanoparticles with Antitumor Property on Caco-2 Colon Cancer Cells. Int. J. Biol. Macromol. 2015, 79, 748–755. 10.1016/j.ijbiomac.2015.05.036. 26051341

[ref58] Savadekar N. R.; Karande V. S.; Vigneshwaran N.; Bharimalla A. K.; Mhaske S. T. Preparation of Nano Cellulose Fibers and Its Application in Kappa-Carrageenan Based Film. Int. J. Biol. Macromol. 2012, 51 (5), 1008–1013. 10.1016/j.ijbiomac.2012.08.014. 22940239

[ref59] Numata Y.; Yamada C.; Kishimoto R.; Kono H. Fabrication and Characterization of Bacterial Cellulose/κ-Carrageenan Composite Sheets. Cellulose 2024, 31 (9), 5623–5634. 10.1007/s10570-024-05868-y.

[ref60] Jin W.; Yu Y.; Hou W.; Wang G.; Zhu Z.; He J.; Cheng S.; Huang Q. Molecular Characteristics of Kappa-Selenocarrageenan and Application in Green Synthesis of Silver Nanoparticles. Int. J. Biol. Macromol. 2019, 141, 529–537. 10.1016/j.ijbiomac.2019.09.016. 31493457

[ref61] Hebeish A. A.; El-Rafie M. H.; Abdel-Mohdy F. A.; Abdel-Halim E. S.; Emam H. E. Carboxymethyl Cellulose for Green Synthesis and Stabilization of Silver Nanoparticles. Carbohydr. Polym. 2010, 82 (3), 933–941. 10.1016/j.carbpol.2010.06.020.

[ref62] Azizi S.; Mohamad R.; Abdul Rahim R.; Mohammadinejad R.; Bin Ariff A. Hydrogel Beads Bio-Nanocomposite Based on Kappa-Carrageenan and Green Synthesized Silver Nanoparticles for Biomedical Applications. Int. J. Biol. Macromol. 2017, 104, 423–431. 10.1016/j.ijbiomac.2017.06.010. 28591593

[ref63] Hadrup N.; Sharma A. K.; Loeschner K. Toxicity of Silver Ions, Metallic Silver, and Silver Nanoparticle Materials after in Vivo Dermal and Mucosal Surface Exposure: A Review. Regul. Toxicol. Pharmacol. 2018, 98, 257–267. 10.1016/j.yrtph.2018.08.007. 30125612

[ref64] Martins A. F.; Vlcek J.; Wigmosta T.; Hedayati M.; Reynolds M. M.; Popat K. C.; Kipper M. J. Chitosan/Iota-Carrageenan and Chitosan/Pectin Polyelectrolyte Multilayer Scaffolds with Antiadhesive and Bactericidal Properties. Appl. Surf. Sci. 2020, 502, 144282 10.1016/j.apsusc.2019.144282.

[ref65] Machado B. R.; Facchi S. P.; de Oliveira A. C.; Nunes C. S.; Souza P. R.; Vilsinski B. H.; Popat K. C.; Kipper M. J.; Muniz E. C.; Martins A. F. Bactericidal Pectin/Chitosan/Glycerol Films for Food Pack Coatings: A Critical Viewpoint. Int. J. Mol. Sci. 2020, 21 (22), 8663 10.3390/ijms21228663. 33212884 PMC7698469

[ref66] Irshad A.; Sarwar N.; Sadia H.; Malik K.; Javed I.; Irshad A.; Afzal M.; Abbas M.; Rizvi H. Comprehensive Facts on Dynamic Antimicrobial Properties of Polysaccharides and Biomolecules-Silver Nanoparticle Conjugate. Int. J. Biol. Macromol. 2020, 145, 189–196. 10.1016/j.ijbiomac.2019.12.089. 31838065

[ref67] Tang S.; Zheng J. Antibacterial Activity of Silver Nanoparticles: Structural Effects. Adv. Healthcare Mater. 2018, 7 (13), 1701503 10.1002/adhm.201701503. 29808627

