# Supplementary material for: Optimized Incorporation of Silver Nanoparticles onto Cotton Fabric Using k-Carrageenan Coatings for Enhanced Antimicrobial Properties
Source: ACS Appl Bio Mater. 2024 Sep 24;7(10):6908–18. doi: 10.1021/acsabm.4c01002 (PMC11497209; doi:10.1021/acsabm.4c01002)
Supplement: Supplementary file 1 — mt4c01002_si_001.pdf [file mt4c01002_si_001.pdf]

Supporting Information

Optimized Incorporation of Silver Nanoparticles onto Cotton Fabric Using k-Carrageenan Coatings for Enhanced Antimicrobial Properties

Luana Dumas[1], Matheus Cardoso de Souza[1], Elton Guntendorfer Bonafe[1], Alessandro Francisco Martins[1,2], Johny Paulo Monteiro[1,*]

[1]Laboratory of Materials, Macromolecules and Composites (LAMMAC), Federal University of Technology - Paraná (UTFPR), Apucarana, PR 86812-460, Brazil.

[2]Department of Chemistry, Pittsburg State University, Pittsburg, KS 66762, USA.

*e-mail address: johnymonteiro@utfpr.edu.br

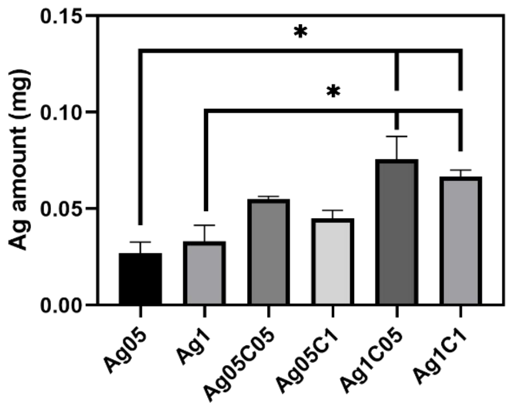

Figure S1. Measurements by FAAS of the incorporated silver amount on cotton fabric from each tested coating. The means values were obtained from triplicate analyses, and * represents significant variations determined from the Tukey test ($p<0.05$).

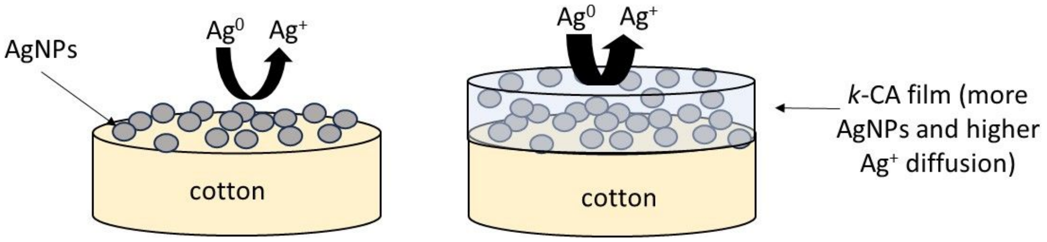

Figure S2. Illustrative scheme for incorporating AgNPs on cotton throughout the volume and limited to the surface for the k-CA/AgNPs-coated and AgNPs-coated samples, respectively. A higher Ag^+ ions diffusive power for k-CA/AgNPs-coated samples.

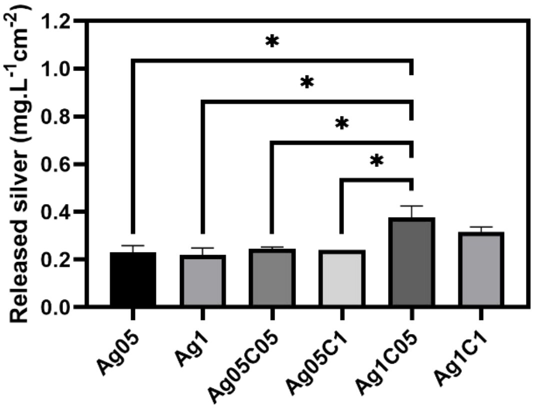

Figure S3. Measurements by FAAS of the released Ag^+ concentration from each coated cotton fabric in the release test. The means values normalized by area (cm^2) were obtained from triplicate analyses, and * represents significant variations determined from the Tukey test ($p<0.05$).
